# The versatile nature of miR-9/9^*^ in human cancer

**DOI:** 10.18632/oncotarget.24889

**Published:** 2018-04-17

**Authors:** Katarzyna Nowek, Erik A.C. Wiemer, Mojca Jongen-Lavrencic

**Affiliations:** ^1^ Department of Hematology, Erasmus MC Cancer Institute, Erasmus University Medical Center, Rotterdam, The Netherlands; ^2^ Department of Medical Oncology, Erasmus MC Cancer Institute, Erasmus University Medical Center, Rotterdam, The Netherlands

**Keywords:** miRNA, miR-9, miR-9^*^, human cancer, miRNA-based therapies

## Abstract

miR-9 and miR-9^*^ (miR-9/9^*^) were first shown to be expressed in the nervous system and to function as versatile regulators of neurogenesis. The variable expression levels of miR-9/9^*^ in human cancer prompted researchers to investigate whether these small RNAs may also have an important role in the deregulation of physiological and biochemical networks in human disease. In this review, we present a comprehensive overview of the involvement of miR-9/9^*^ in various human malignancies focusing on their opposing roles in supporting or suppressing tumor development and metastasis. Importantly, it is shown that the capacity of miR-9/9^*^ to impact tumor formation is independent from their influence on the metastatic potential of tumor cells. Moreover, data suggest that miR-9/9^*^ may increase malignancy of one cancer cell population at the expense of another. The functional versatility of miR-9/9^*^ emphasizes the complexity of studying miRNA function and the importance to perform functional studies of both miRNA strands in a relevant cellular context. The possible application of miR-9/9^*^ as targets for miRNA-based therapies is discussed, emphasizing the need to obtain a better understanding of the functional properties of these miRNAs and to develop safe delivery methods to target specific cell populations.

## INTRODUCTION

MiRNAs are short non-coding RNAs that by binding to target mRNAs decrease protein levels and in this way regulate crucial cellular processes. [1–3] miRNA transcripts are expressed as hairpin-like precursor structures that undergo stepwise maturation into double-stranded miRNA/miRNA^*^ duplexes. In the past, it was proposed that one of the strands, called the mature miRNA, is stabilized and becomes functional, whereas another, referred to as the passenger strand or miRNA^*^, is degraded. Recently, it has been shown that miRNA^*^s can also display functionality and play complementary roles to their related miRNAs. [4–6]

miR-9 (miR-9-5p) and miR-9^*^ (miR-9-3p) are two miRNAs that originate from the same precursor and are highly conserved during evolution from flies to humans. [7] All vertebrate miR-9/9^*^ orthologs have an identical mature sequence. In mammals, miR-9/9^*^ are encoded by three genes: *MIR9-1*, *MIR9-2* and *MIR9-3*. In humans, these genes are located on the chromosomes 1 (1q22), 5 (5q14.3) and 15 (15q26.1), respectively. miR-9/9^*^ are mainly expressed in the nervous system and were initially studied as regulators of neurogenesis. [8] Interestingly, aberrant expression of miR-9/9^*^ has been found in various types of human cancer revealing an unanticipated functional versatility. [9–11] The high level of sequence conservation and the fact that miR-9/9^*^ are encoded by three different genomic loci points to important functional roles of these miRNAs that may be exploited by cancer cells.

In the past years, several studies have reported on the relationship of miR-9/9^*^ expression with different cellular processes, such as differentiation, proliferation, migration and metastasis. [11–14] Interestingly, miR-9 and miR-9^*^, although concomitantly expressed from one precursor miRNA, may be preferentially retained and can play synergistic or opposite roles within one malignancy. [15–17] Here, we summarize the diverse functions of miR-9/9^*^ in the biology of human cancer. We outline the mechanisms through which miR-9/9^*^ are involved in tumorigenesis and the cellular context in which these miRNAs operate. Although most of the reported findings still need validation under physiological (*in vivo*) conditions, they underscore the complexity of miRNA functionality within the heterogeneous population of cancer cells. This review may serve as the basis for a broader dispute about the often counteracting functions of a particular miRNA in the pathobiology of human cancer and their implications for future treatment opportunities.

## GLIOBLASTOMA MULTIFORME

Glioblastoma multiforme (GBM; grade IV astrocytoma) is the most common and aggressive brain tumor. [18] It has been proposed that GBM originates from the cancer cell population with stem cell-like properties that is characterized by CD133 expression. [19] GBM can be divided into clinically and genetically distinct groups based on the similarity of miRNA and mRNA expression signatures to different neural precursor cell types: radial glia, oligoneuronal precursors, neuronal precursors, neuroepithelial/neural crest precursors or astrocyte precursors. [20]

**Figure 1:**
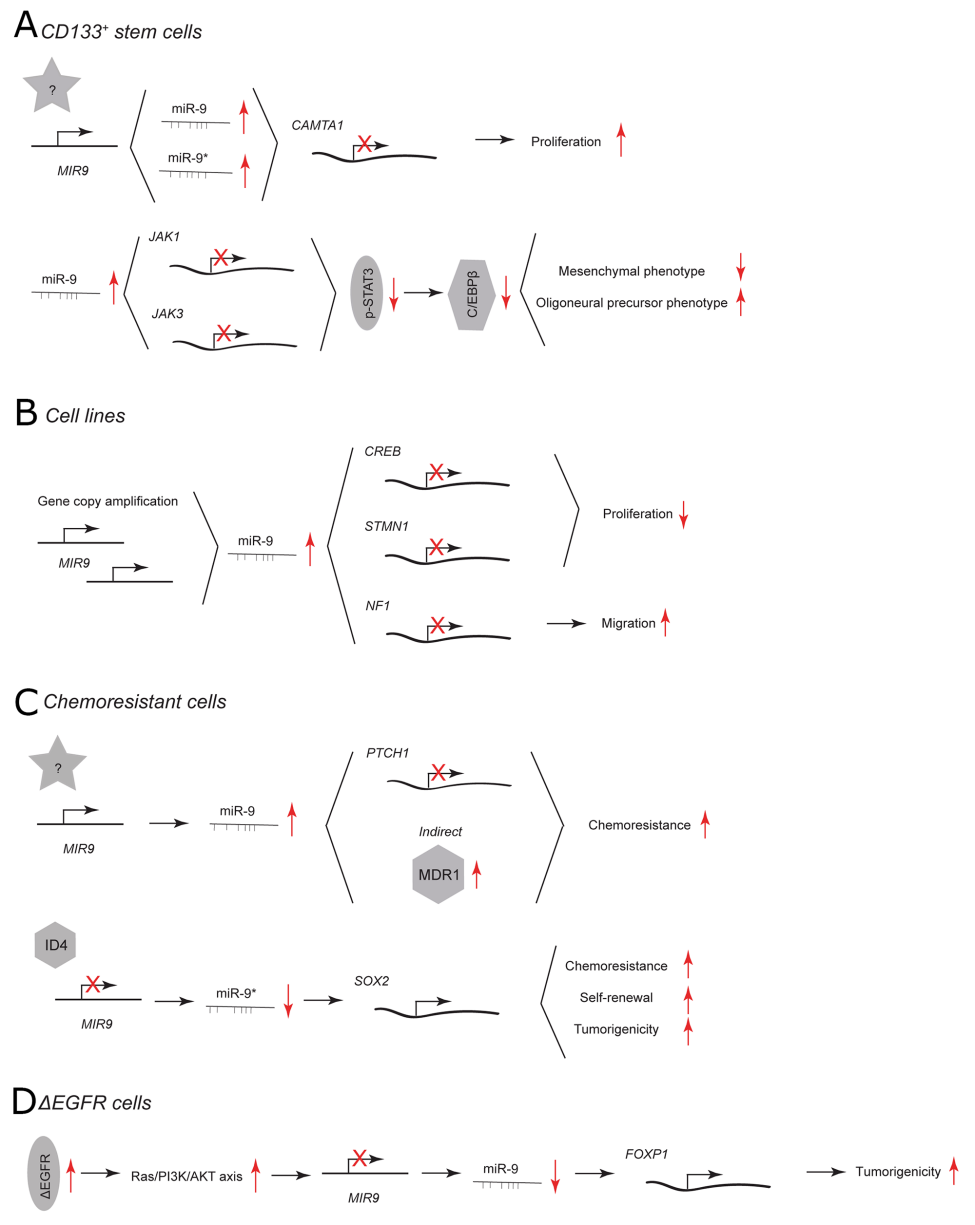
miR-9 and miR-9^*^ functions in human glioblastoma multiforme Each graph schematically depicts the reported levels of expression of miR-9/9^*^ as well as their functional significance including relevant target genes and phenotypical effects in **(A)** CD133^+^ stem cells, **(B)** glioblastoma cell lines, **(C)** chemoresistant glioblastoma cells, **(D)** ΔEGFR cells.

In CD133^+^ GBM stem cells, miR-9/9^*^ are highly expressed and needed for stem cell renewal. [17] Inhibition of miR-9 as well as miR-9^*^ using 2’-*O*-methylated antisense inhibitors results in reduced colony numbers (Figure 1A). Both miRNAs directly target a tumor suppressor calmodulin binding transcription activator 1 (CAMTA1), of which overexpression mimics the phenotype of miR-9/9^*^ inhibition. Additionally, R28 GBM cells that overexpress CAMTA1 form smaller tumors *in vivo* than control cells.

The highest expression of miR-9 has been found in the oligoneural subclass of GBM. [20] miR-9 is considered a regulator of a subtype-specific gene expression network and drives subtype-specific cell decisions. [20] Overexpression of miR-9 using a mimic in CD133^+^ GBM stem cells promotes oligoneural and suppresses a more aggressive mesenchymal phenotype by downregulating expression of Janus kinases (JAK1 and JAK3), inhibiting activation of signal transducer and activator of transcription 3 (STAT3) and decreasing expression of the STAT3 transcriptional target CCAAT/enhancer-binding protein β (C/EBPβ) (Figure 1A). [20, 21]

In GBM cell lines, miR-9 has been reported to play a critical role in determination of the so-called “go or grow” phenotype. [13] miR-9 is part of a feedback minicircuitry that allows a tight control of the expression levels of target genes that coordinate the proliferation and migration of GBM cells (Figure 1B). In contrast to increasing colony numbers of CD133^+^ GBM stem cells via CAMTA1, miR-9 has been shown to inhibit proliferation of GBM cell lines by targeting the cyclic AMP response element-binding protein (CREB) but to promote migration by targeting neurofibromin 1 (NF1). Additionally, the transcription of both miR-9 and NF1 is under CREB’s control. Gene copy amplification of miR-9 hinders the balance of this regulatory minicircuitry and contributes to motility of GBM cells. Another miR-9 target that contributes to reduced proliferation and tumor growth is stathmin (STMN1), which regulates microtubule formation dynamics during cell-cycle progression. [22, 23] U87MG GBM cells transfected with miR-9 mimic are characterized by decreased expression of STMN1 and form smaller tumors than control cells.

In GBM cells that are resistant against alkylating agents, miR-9 is highly expressed and miR-9^*^ is downregulated. [15, 16, 24, 25] miR-9 has been shown to contribute to the chemoresistance of GBM cells by direct targeting of patched homolog 1 protein (PTCH1) and subsequent activation of sonic hedgehog (SHH) signaling pathway (Figure 1C). [25] Additionally, the delivery of anti-miR-9 to the resistant GBM cells indirectly downregulates the expression of the multidrug transporter (MDR1) and sensitizes the GBM cells to chemotherapy. [15] miR-9^*^ is part of an ID4-miR-9^*^-SOX2-ABCC3/ABCC6 regulatory pathway. [16] Inhibitor of differentiation 4 (ID4) suppresses miR-9^*^ expression and upregulates the direct target of this miRNA SRY (sex determining region Y)-box 2 (SOX2). SOX2 is highly expressed in patients with GBM. [26] Its upregulation leads to increased chemoresistance, self-renewal and tumorigenicity of GBM cell lines and patient-derived CD133^+^ GBM stem cells. [16]

40% to 50% of primary GBM cases exhibit epidermal growth factor receptor (EGFR) amplification, overexpression, and/or mutations. [27] An EGFR mutant that lacks exons 2-7 (ΔEGFR) is constitutively active and present in a high proportion of GBM cases with EGFR amplification. This EGFR mutant confers a strong tumor-enhancing effect by promoting growth, cell invasion and chemoresistance. [28–30] In GBM cells that express ΔEGFR, miR-9 acts as a tumor suppressor that downregulates transcription factor forkhead box P1 (FOXP1) (Figure 1D). [31] Viral overexpression of miR-9 or silencing of FOXP1 antagonizes ΔEGFR-dependent tumor growth *in vivo*. ΔEGFR activates Ras/PI3K/AKT, which in turn suppresses miR-9. Of note, the viral transduction as used here likely results in overexpression of both miR-9 and miR-9^*^ making it difficult to discern whether both or only a single miRNA display activity. However, as the presented outcome is in line with the previously mentioned reports concerning the function of miR-9^*^ in chemoresistant GBM cells the expression of miR-9^*^ and its influence on tumorigenicity of ΔEGFR GBM cells needs to be further investigated. [16]

## BREAST CANCER

Breast cancer (BC) is a heterogeneous malignancy that can be classified by estrogen receptor (ESR1) expression (ER^+^), human epidermal growth factor receptor 2 (ERBB2) expression (HER2^+^), the absence of ESR1, ERBB2 and the progesterone receptor in triple-negative breast cancer (TNBC) or the expression of driver oncogenes (e.g. MYC). [32–35] A vast amount of data concerning the diverse roles of miR-9/9^*^ have been obtained for breast cancer.

**Figure 2:**
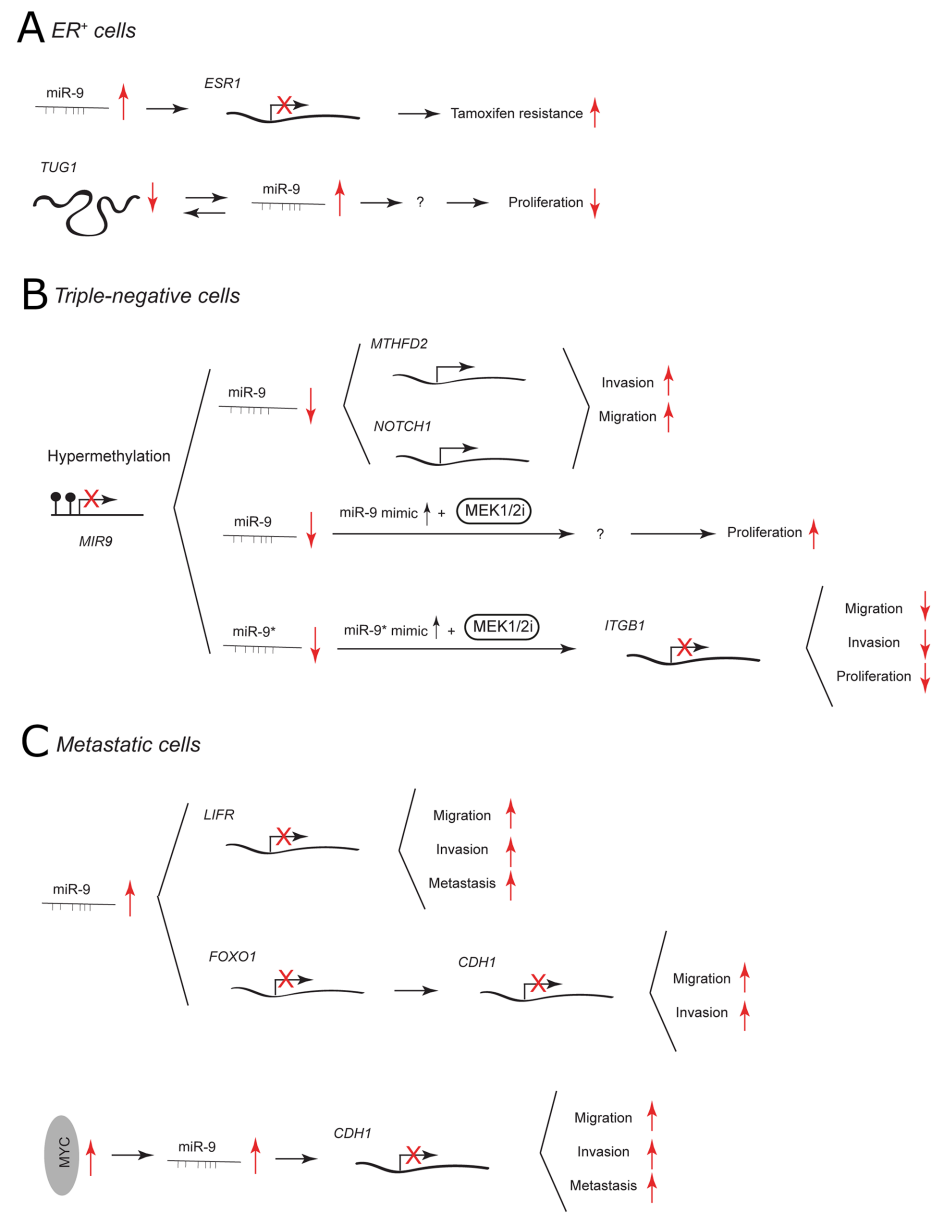
miR-9 and miR-9^*^ functions in human breast cancer Each graph schematically depicts the reported levels of expression of miR-9/9^*^ as well as their functional significance including relevant target genes and phenotypical effects in **(A)** ER^+^ cells, **(B)** triple-negative cells, **(C)** metastatic cells.

Because of the availability of endocrine-targeted therapy (e.g. tamoxifen treatment), patients with BC that express ER have better prognosis. [36] Nonetheless, therapeutic resistance eventually occurs in a large number of cases. In the ER^+^ MCF-7 cell line, miR-9 has been shown to directly target ER and to influence, not only ER signaling but also other steroid receptor pathways (Figure 2A). [37] miR-9 levels are reduced in most of ER^+^ BC cases compared to ER^-^. However, when upregulated it is associated with worse patient outcome and its viral overexpression in MCF-7 cells contributes to tamoxifen resistance. [37, 38] The expression of miR-9 in ER^+^ BC has recently been linked to the level of lncRNA taurine-upregulated gene 1 (*TUG1*). It has been proposed that *TUG1* and miR-9 may co-regulate each other to impact cell proliferation [39].

In TNBC cells, miR-9/9^*^ are expressed at low levels due to promoter hypermethylation of the *MIR-9* loci. [40] miR-9 has been suggested to play a tumor suppressive role by targeting mitochondrial bifunctional enzyme MTHFD2 and NOTCH1 receptor (Figure 2B). [14, 41] Overexpression using pre-miR-9 or lentiviral constructs decreases the invasiveness and migration of TNBC MDA-MB-231 cells. [14, 41] In line with this, knockdown of MTHFD2 recapitulates the anti-invasive effect of miR-9. NOTCH1 is known to be involved in the pathogenesis of TNBC and its inhibition reduces the migratory potential of MDA-MB-231 cells. [42, 43] Interestingly, the downregulation of NOTCH1 with γ-secretase inhibitors in ER^+^ MCF-7 cell line stimulates migration *in vitro* and promotes tumor growth *in vivo*. [43] Recently, it has been reported that miR-9 may influence TNBC aggressiveness by taking part in cross-talk between cancer cells and cancer-associated fibroblasts [44].

Mitogen-activated protein kinase enzymes 1 and 2 (MEK1/2) inhibitors have been used in cancer therapy but can become ineffective due to acquired drug resistance. [45] In TNBC cells, treatment with a MEK1/2 inhibitor together with a miR-9 mimic increases cell proliferation, whereas treatment together with a miR-9^*^ mimic suppresses growth, migration and invasion of tumor cells (Figure 2B). [40] miR-9^*^ activity is mediated through downregulation of β_1_ integrin(ITGB1), which is important for growth factor receptor and extracellular matrix-related signaling.

The expression of miR-9 has been widely related to BC metastasis. In non-metastatic SUM159 cells, miR-9-mediated downregulation of leukemia inhibitory factor receptor (LIFR) induces migration, invasion and metastatic colonization through deregulation of the Hippo-YAP pathway. [46] Additionally, miR-9 has been reported to be higher expressed in metastatic than in non-metastatic primary human breast cancer. In MCF-7 and MDA-MB-231 cells, miR-9 has been shown to downregulate the expression of another tumor suppressor gene FOXO1 that belongs to the FOXO family of Forkhead transcription factors. [47] FOXO1 3’ UTR may sequester miR-9 from E-cadherin 3’ UTR. Overexpression of FOXO1 leads to upregulation of E-cadherin and decreases the migration and invasiveness of BC cell lines. In 2010, Ma *et al.* reported that miR-9 plays an important role in metastasis of MYC-driven breast tumors. [11] MYC oncoprotein activates miR-9 expression, which consequently causes downregulation of miR-9 direct target E-cadherin (Figure 2C). This leads to increased cell motility and invasiveness of BC cells *in vitro*. E-cadherin is an epithelial cell adhesion molecule that forms the core of adherens junctions between adjacent epithelial cells and its inactivation enables dissociation of carcinoma cells. [48] By targeting E-cadherin in breast tumor cells, miR-9 enables non-metastatic cells to form pulmonary micrometastasis. [11] In summary, the data show that in BC miR-9 can target two alternative metastatic suppressors: LIFR (which activates Hippo signaling, leading to inactivation of the transcriptional co-activator YAP) and E-cadherin (that maintains adherens junctions) [11, 46].

## CERVICAL CANCER

**Figure 3:**
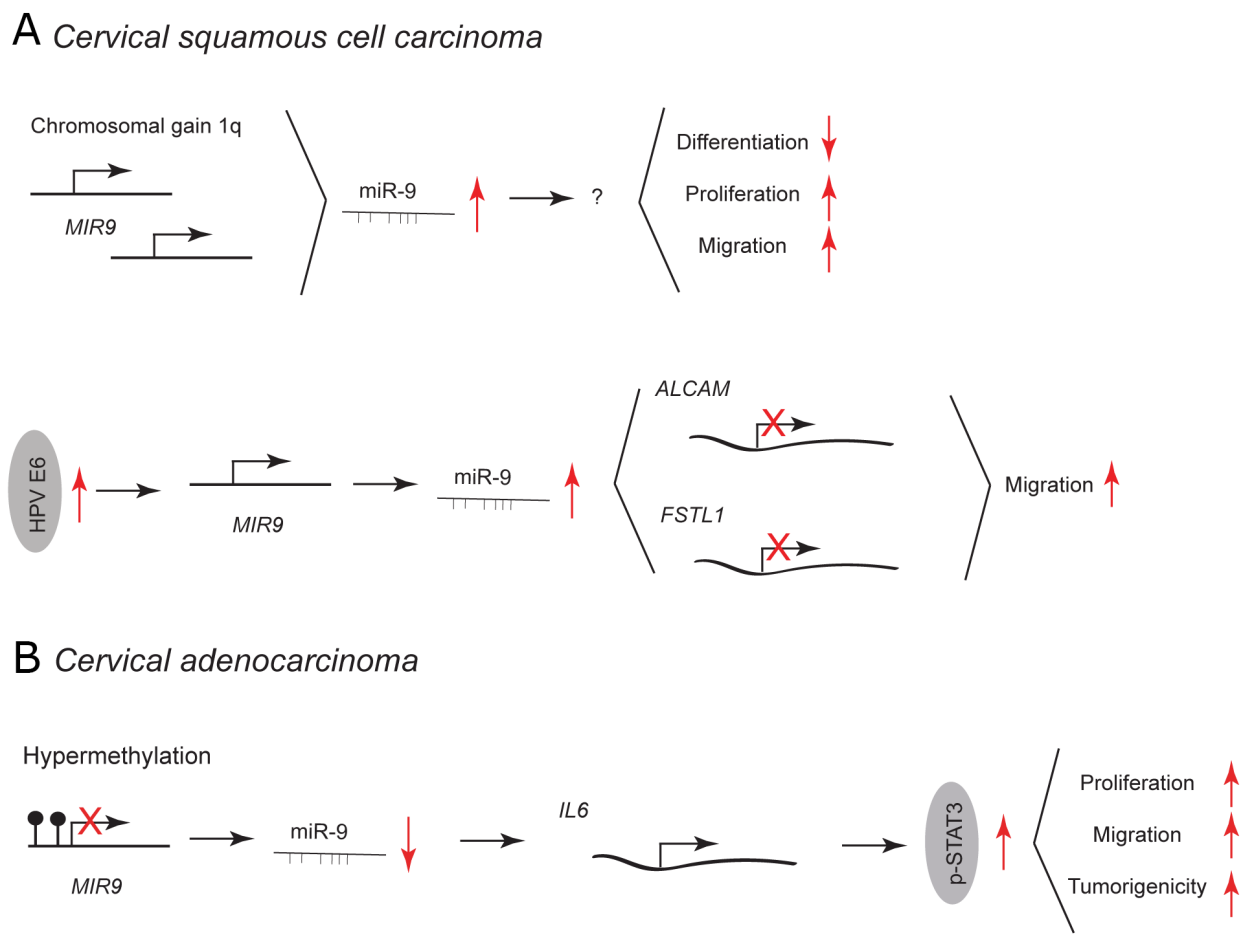
miR-9 and miR-9^*^ functions in human cervical cancer Each graph schematically depicts the reported levels of expression of miR-9/9^*^ as well as their functional significance including relevant target genes and phenotypical effects in **(A)** cervical squamous cell carcinoma cells, **(B)** cervical adenocarcinoma cells.

Cervical cancer can be classified into two prevailing subtypes: cervical squamous cell carcinoma (CSCC; about 80% of cases) and cervical adenocarcinoma (CA; about 5-20% of cases). [49] In CSCC, a chromosomal gain of 1q results in upregulation of miR-9 (1q23.3) and is linked with malignant progression (Figure 3A). [50] Overexpression of miR-9 in normal keratinocytes blocks epithelial differentiation, and induces proliferation and migration. Beside chromosomal gain, an elevated expression of miR-9 in CSCC is caused by human papillomavirus (HPV) infection (Figure 3A). [51] miR-9 expression is activated by HPV E6 – an essential oncogene in cervical cancer development. In normal keratinocytes, overexpression of HPV E6 and miR-9 leads to downregulation of miR-9 target genes involved in cell migration, such as activated leukocyte cell adhesion molecule (ALCAM) and follistatin-related protein 1 (FSTL1). [51–53] This leads to increase in cell motility [51].

In CA, miR-9 is downregulated due to frequent promoter-hypermethylation and has been shown to act as a tumor suppressor (Figure 3B). [54] Ectopic expression of miR-9 inhibits the JAK/STAT3 pathway by targeting interleukin 6 (IL-6). This results in decreased proliferation and migration of HeLa cells *in vitro* and reduced tumor growth *in vivo*. IL-6 is highly expressed in human cervical cancer promoting tumorigenesis by activation of the JAK/STAT3 pathway, subsequent upregulation of vascular endothelial growth factor (VEGF) and increased angiogenesis [55].

## SQUAMOUS CELL CARCINOMA OF SKIN AND ORAL CAVITY

**Figure 4:**
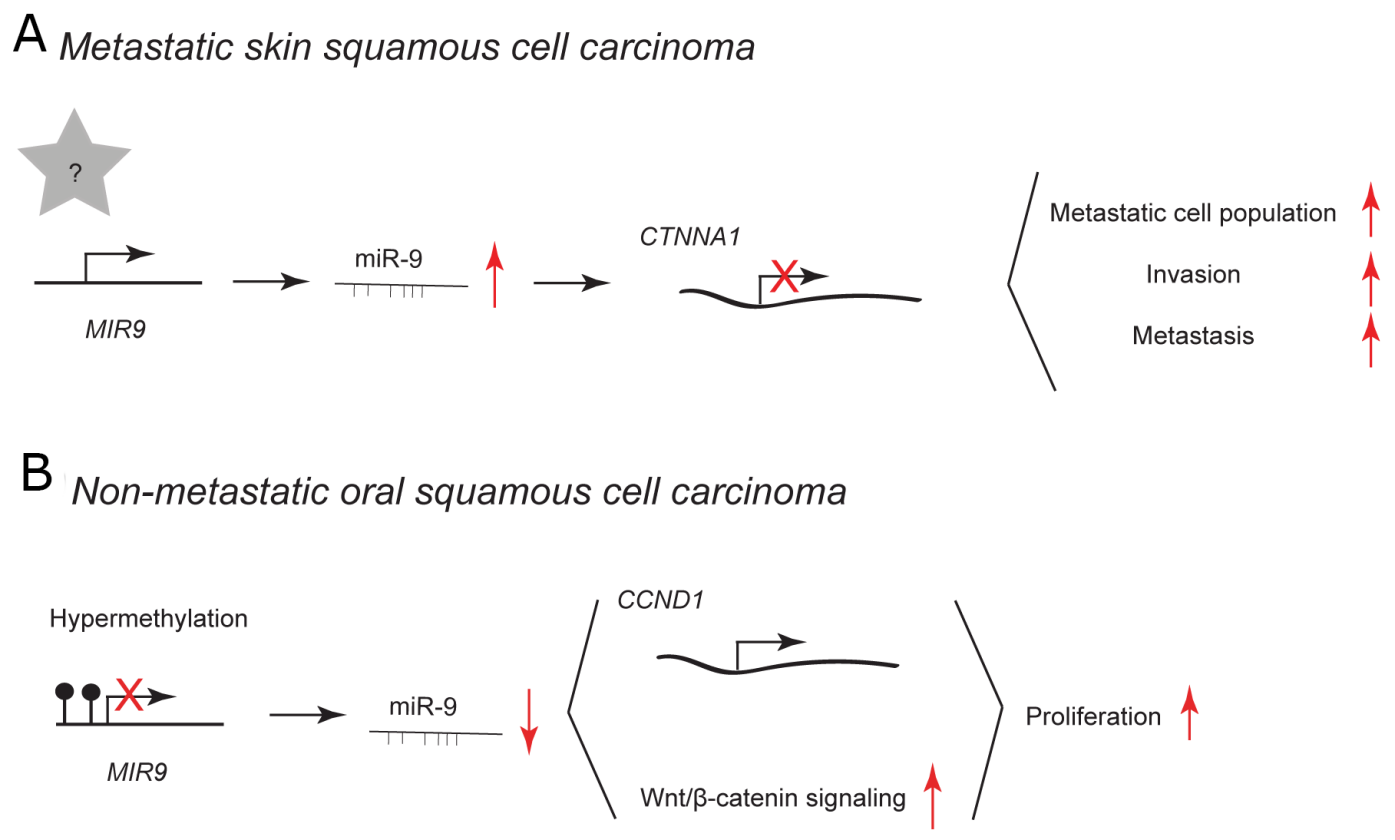
miR-9 and miR-9^*^ functions in human skin and oral cavity squamous cell carcinoma Each graph schematically depicts the reported levels of expression of miR-9/9^*^ as well as their functional significance including relevant target genes and phenotypical effects in **(A)** metastatic skin squamous cell carcinoma cells, **(B)** non-metastatic oral squamous cell carcinoma cells.

Squamous cell carcinoma (SCC) is a type of cancer that develops from squamous epithelial cells in diverse tissues, e.g. within skin and oral cavity. Cells of skin epithelium undergo constant self-renewal throughout life, therefore it is believed that SCC originates from keratin 15-expressing stem cells (K15^+^) that harbor pro-proliferative mutations in *Kras*^G12D^. [56] Additional deletion of *Smad4* in these cells leads to the spontaneous development of multi-lineage tumors, including metastatic squamous cell carcinoma. [57, 58] In murine *K15.Kras*^*G12D*^*.Smad4*^*–/–*^ cancer stem cell-enriched population, viral overexpression of miR-9 leads to the expansion of metastatic cell population resulting in increased invasion and metastasis (Figure 4A). [58] In primary human SCC cells, high expression of miR-9 correlates with metastasis and the loss of a predicted direct target α-catenin. However, α-catenin depletion alone does not cause SCC metastasis suggesting that additional targets are required for miR-9-mediated effect. [59] miR-9 has been reported to be expressed at high levels in patients with recurrent head and neck SCC [60].

In non-metastatic human oral SCC specimens, miR-9 is downregulated probably due to frequent promoter hypermethylation. [61, 62] Overexpression using miR-9 mimic in human the UM-SCC22A cell line inhibits cell proliferation (Figure 4B). [61] Curcumin has been reported to have growth-suppressive potential in different types of cancer, as well as in oral SCC. [62, 63] In the human SCC-9 cell line, curcumin treatment leads to upregulation of miR-9, which in turn inhibits cell proliferation via downregulation of cyclin D1 and suppression of Wnt/β-catenin signaling (Figure 4B). [62] Cyclin D1 and the Wnt/β-catenin signaling pathway are frequently deregulated in human cancer and may play essential roles in the process of tumorigenesis [64, 65].

## HEMATOLOGICAL MALIGNANCIES

Hematopoiesis is a hierarchical differentiation process in which hematopoietic stem cells (HSCs) undergo step-wise maturation into various types of blood cells. [66, 67] During this process, HSCs lose their self-renewal and multi-lineage differentiation capability to give rise to lymphoid and myeloid progeny. Deregulation of normal hematopoiesis may result in development of hematological tumors. [68, 69] Acute and chronic myelogenous leukemia, myelodysplastic syndromes, and myeloproliferative disorders are tumors derived from the myeloid line, whereas lymphomas, lymphocytic leukemias, and myeloma have a lymphoid origin. Hematological malignancies are heterogeneous disorders that are characterized by frequent chromosomal abnormalities, genetic mutations and aberrations in epigenetic regulation. [68, 69]

**Figure 5:**
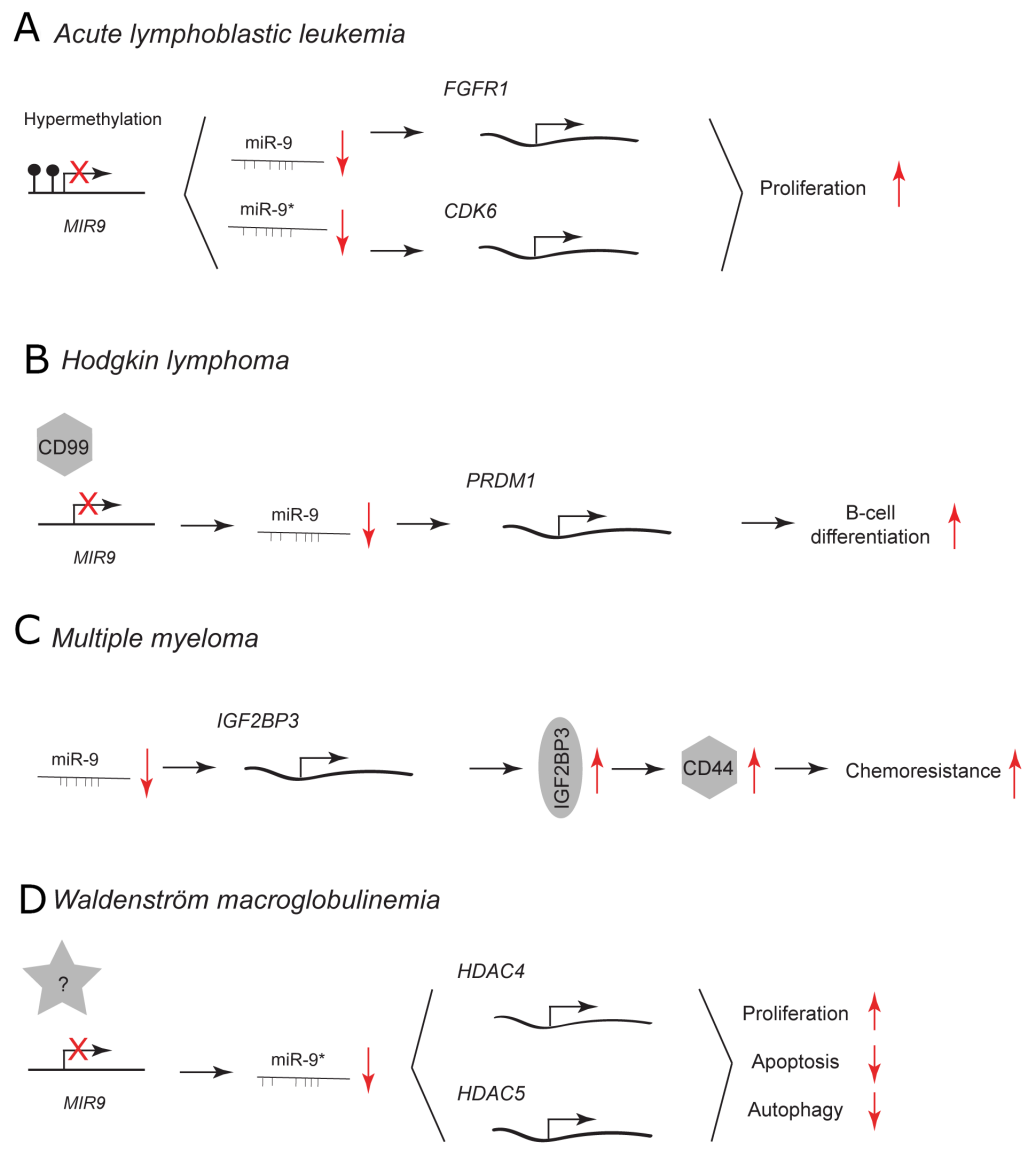
miR-9 and miR-9^*^ functions in human lymphoid malignancies Each graph schematically depicts the reported levels of expression of miR-9/9^*^ as well as their functional significance including relevant target genes and phenotypical effects in **(A)** acute lymphoblastic leukemia cells, **(B)** Hodgkin lymphoma cells, **(C)** multiple myeloma cells, **(D)** Waldenström macroglobulinemia cells.

In acute lymphoblastic leukemia (ALL), low miR-9 expression is associated with hypermethylation of *MIR9* gene family (Figure 5A). [70] This epigenetic downregulation leads to upregulation of predicted miR-9 and miR-9^*^ targets, fibroblast growth factor receptor 1 (FGFR1) and cyclin-dependent kinase 6 (CDK6). FGFR1 and CDK6 are involved in cell proliferation and survival. [71, 72] Treatment with FGFR1 and CDK6 inhibitors suppresses the proliferation of ALL cells. [70] *MIR9* genes have been reported to be also frequently methylated in chronic lymphocytic leukemia (CLL) and overexpression of miR-9 using a mimic decreases CLL cell proliferation. [73]

CD99 is a transmembrane glycoprotein that is implicated in cell migration, adhesion and differentiation. [74–76] It is expressed at low levels in Hodgkin/Reed-Sternberg (HRS) cells of Hodgkin lymphoma (HL). [77] CD99 downregulates the expression of miR-9 and upregulates a direct miR-9 target: positive regulatory domain 1 (PRDM1/BLIMP-1) (Figure 5B). [10, 77] PRDM1 is the master regulator of terminal B-cell differentiation. miR-9 is highly expressed in HL cells and its downregulation by CD99 overexpression or a direct knockdown using miR-9 inhibitor augments PRDM1 levels that trigger B-cell differentiation into plasma cells. [77] During normal B-cell development within the germinal centers, B cells closely interact with follicular dendritic cells (FDC). [78] Only B cells that bind to these cells survive in the germinal centers and differentiate. It has been shown that direct cell-cell contact between follicular dendritic cells and B cells leads to downregulation of miR-9 and upregulation of PRDM1. This subsequently may promote B-cell differentiation.

In multiple myeloma (MM), insulin-like growth factor 2 mRNA binding protein 3 (IGF2BP3) stabilizes the expression of a cell surface glycoprotein CD44 that is involved in drug resistance of MM cells. [79] Histone deacetylase (HDAC) inhibitors are promising novel chemotherapeutics in MM since they downregulate CD44 expression. HDAC inhibitors treatment leads to upregulation of miR-9 and downregulation of its direct target IGF2BP3 (Figure 5C). Subsequent downregulation of CD44 sensitizes the resistant MM cell to lenalidomide treatment.

miR-9^*^, has been reported to have a tumor suppressive role in Waldenström macroglobulinemia (WM) (Figure 5D). [80] WM is a B-cell low-grade lymphoma characterized by the accumulation of B cells in the bone marrow. miR-9^*^ is expressed at reduced levels in WM CD19^+^ cells compared to normal CD19^+^ counterparts. Its overexpression using pre-miR-9^*^ in WM cells inhibits the unbalanced HDAC activity by downregulation of HDAC4 and 5. This results in decreased proliferation, increased apoptosis and autophagy. Neither adherence to primary BM stromal cells nor growth factors protected against the miR-9^*^-dependent growth inhibition. Aberrant HDAC activity has been reported to have a tumorigenic effect in many malignancies by influencing the expression of genes controlling cellular proliferation, differentiation and apoptosis [81].

**Figure 6:**
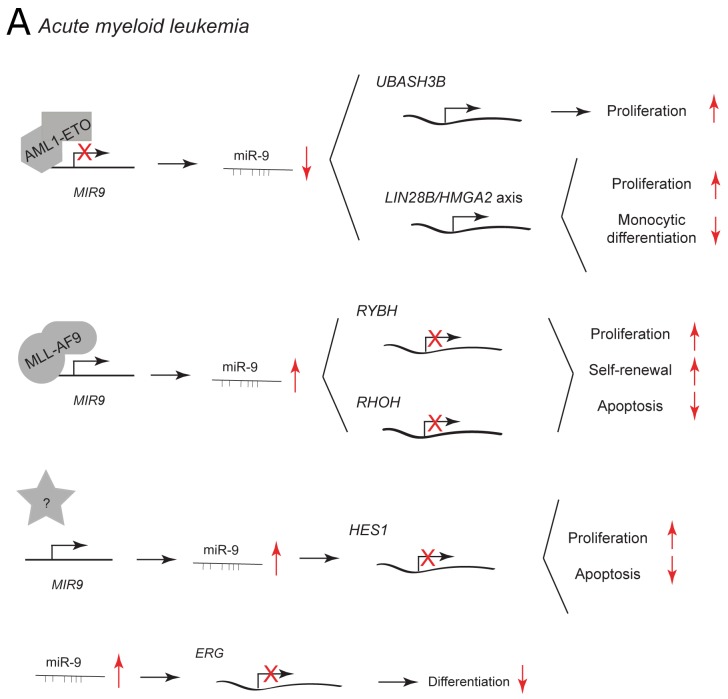
miR-9 and miR-9^*^ functions in human myeloid malignancies Each graph schematically depicts the reported levels of expression of miR-9/9^*^ as well as their functional significance including relevant target genes and phenotypical effects in **(A)** acute myeloid leukemia cells.

In acute myeloid leukemia (AML), miR-9 has been reported to be differentially expressed between AML subtypes. [12, 82, 83] Dependent on the type of leukemic cell, it may suppress or promote leukemic development. The t(8;21) rearrangement is the most common chromosomal translocation in AML resulting in the formation of AML1-ETO fusion protein. [84] AML1-ETO downregulates miR-9 and in this way promotes the expression of *UBASH3B*/Sts-1, a tyrosine phosphatase that inhibits CBL and enhances STAT5/AKT/ERK/Src signaling to promote myeloid proliferation (Figure 6A). Ectopic expression of miR-9 in t(8;21) AML cells reduces leukemic growth and enhances monocytic differentiation induced by calcitrol by direct repression of the oncogenic LIN28B/HMGA2 axis. [82] LIN28 and HMGA2 are expressed in undifferentiated proliferating cells during embryogenesis and their upregulation in adult cells leads to oncogenic transformation [85, 86].

miR-9 is highly upregulated in *MLL*-rearranged leukemic cells as compared to non-*MLL*-rearranged cells and normal controls (Figure 6A). [12, 83] MLL fusion proteins may promote miR-9 expression by direct binding to the promoter regions of *MIR9* genes. Knockdown of endogenous miR-9 expression with a miR-9 sponge inhibits MLL fusion–induced immortalization/transformation of normal hematopoietic progenitor cell, whereas its viral overexpression has the opposite effect. miR-9 function may be mediated by the two predicted targets: RING1 and YY1-binding protein (RYBH) and Ras homolog family member H (RHOH). RYBP is a polycomb complex-associated protein that can stabilize p53 and has tumor suppressor activity. [87] RHOH is a member of the Rho GTPase protein family and it can function as an oncogene or tumor suppressor depending on the context [88].

In AML patients with a normal karyotype, miR-9 is expressed at higher levels in leukemic stem/progenitor cells (LSPCs) than in normal hematopoietic stem cells derived from the same patient. [89] Additionally, miR-9 expression is inversely correlated to the levels of hairy and enhancer of split-1 (HES1), a known tumor-suppressor (Figure 6A). [90, 91] Knockdown of miR-9 by lentiviral infection decreases leukemic cell proliferation and survival by increasing HES1 expression *in vitro* and *in vivo* [89].

miR-9/9^*^ are both aberrantly upregulated in most of human AML cases. [12] In normal hematopoietic stem and progenitor cells, ectopic expression of miR-9/9^*^ inhibits myeloid differentiation by post-transcriptional regulation of ETS-related gene (ERG) (Figure 6A). ERG is a transcription factor that is essential for definitive hematopoiesis and its functional activity depends on its expression level. [12, 92, 93] In patients with AML, expression of miR-9 has no prognostic significance, whereas miR-9^*^ predicts favorable outcome. [94] Recently, it has been proposed that miR-9^*^ may sensitize tumor cells to chemotherapy in chronic myelogenous leukemia [95].

## CONCLUSIONS AND OUTLOOK

Initially discovered as versatile regulators of neurogenesis, miR-9/9^*^ quickly became a focus of attention in cancer research. In the past years, multiple studies have reported on the deregulated expression of miR-9/9^*^ in various types of human cancer and the relation of their aberrant expression levels with different processes, e.g. self-renewal, proliferation and differentiation. Furthermore, these miRNAs have been shown to have important regulatory roles in cancer biology regulating processes such as tumor initiation, tumor progression and chemosensitivity. Table 1 summarizes the different reported functions of miR-9/9^*^ in various cell and tumor types. It also provides information on the up- or downregulation of miR-9/9^*^ and lists putative mRNA targets and target-related pathways according to www.genecards.org. It is evident that miR-9/9^*^ expression affects many biochemical pathways commonly deregulated in human cancer such as the PI3K/AKT, JAK/STAT, NOTCH1, Wnt/β-catenin, Ras and ERK signaling pathways. This underscores the relevance and intricate involvement of miR-9/9^*^ in human cancer biology. The picture that emerges from the current literature is still fragmentary impeding firm conclusions about the role(s) of miR-9/9^*^ in cancer. More research is needed that incorporates: 1) systems biology to delineate and integrate the miR-9/9^*^ regulatory networks; 2) *in vivo* experiments performed under physiological conditions and 3) the need to address miR-9 and miR-9^*^ functions separately. Interestingly, miR-9 and miR-9^*^ serve as an example of miRNAs that, although co-transcribed and derived from the same precursor, may fulfill different and sometimes opposing functions. As of yet, not much is known about the functional relationship between miR-9 and miR-9^*^ and which factors determine their individual stability and functionality. These insights are critical to improve our understanding of the functional significance of miR-9/9^*^ in the context of cancer.

**Table 1:** Summary of the reported oncogenic or tumor suppressor functions of miR-9 and 9^*^ in human cancer. Tumor types and functions affected are listed in alphabetical order. It is indicated whether miR-9 levels are increased (↑) or decreased (↓) together with a list of direct targets when miR-9 or 9* is expressed or re-introduced in the given cell type. The information about the possible pathways involved has been added according to the literature based on the reported targets.

Function			Apoptosis	Autophagy	Cell frequency	Chemo/drug resistance	Differentiation	Invasion	Metastasis	Migration	Proliferation	Self-renewal	Tumori-genicity
Tumor	Cell type	Feature											
BC	ER^+^	Direction				↑					↓		
		Target				*ESR1*					*TUG1*		
		Pathway^*^				ER signaling ERK							
	Metastatic	Direction						↑	↑	↑			
		Target						*LIFR* *CDH1*	*LIFR* *CDH1* *FOXO1*	*LIFR* *CDH1* *FOXO1*			
		Pathway						Ras ERK E-cadherin	Ras ERK E-cadherin PI3K/AKT	Ras ERK E-cadherin PI3K/AKT			
	TNBC	Direction						↓		↓			
		Target						*MTHFD2* *NOTCH1*		*MTHFD2* *NOTCH1*			
		Pathway						ERK NOTCH1		ERK NOTCH1			
GBM	CD133^+^	Direction					#				↑↑		
		Target					*JAK1* *JAK3*				*CAMTA1* *CAMTA1*		
		Pathway					ERK JAK/STAT EGFR PI3K/AKT						
	Cell lines	Direction								↑	↓		
		Target								*NF1*	*CREB* *STMN1*		
		Pathway								EGFR ERK Ras	NOTCH1 JAK/STAT EGFR ERK		
	Chemo-resistant	Direction				↑↓						↓	↓
		Target				*PTCH1* *SOX2*						*SOX2*	*SOX2*
		Pathway				ERK Wnt						ERK Wnt	ERK Wnt
	ΔEGFR	Direction											↓
		Target											*FOXP1*
		Pathway											Wnt
CC	CA	Direction								↓	↓		↓
		Target								*IL6*	*IL6*		*IL6*
		Pathway								JAK/STAT ERK	JAK/STAT ERK		JAK/STAT ERK
	CSCC	Direction					↓			↑	↑		
		Target								*ALCAM* *FSTL1*			
		Pathway								CAM			
HM	ALL	Direction									↓↓		
		Target									*FGFR1* *CDK6*		
		Pathway									ERK Ras PI3K/AKT		
	AML	Direction	↓				↑↓				↑↓	↑	
		Target	*RYBH* *RHOH* *HES1*				*LIN28B/HMGA2* *ERG*				*UBASH3B* *LIN28B/HMGA2* *RYBH* *RHOH* *HES1*	*RYBH* *RHOH*	
		Pathway	ERK AKT NOTCH1				Wnt				ERK AKT NOTCH1 Wnt	ERK	
	HL	Direction					↓						
		Target					*PRDM1*						
		Pathway					TP53 NF-kappaB						
	MM	Direction				↓							
		Target				*IGF2BP3*							
		Pathway				IGF2BP							
	WM	Direction	↑	↑							↓		
		Target	*HDAC4* *HDAC5*	*HDAC4* *HDAC5*							*HDAC4* *HDAC5*		
		Pathway	JAK/STAT NOTCH1 HDAC	JAK/STAT NOTCH1 HDAC							JAK/STAT NOTCH1 HDAC		
SCC	Oral	Direction									↓		
		Target									*CCND1*		
		Pathway									ERK JAK/STAT AKT Wnt		
	Skin	Direction			↑			↑	↑				
		Target			*CTNNA1*			*CTNNA1*	*CTNNA1*				
		Pathway			Wnt ERK E-cadherin			Wnt ERK E-cadherin	Wnt ERK E-cadherin				

^*^: Possible affected target-related pathways according to www.genecards.org.

^#^: miR-9 has been reported to influence the direction of differentiation – it promotes oligoneural and suppresses more aggressive mesenchymal phenotype.

Functions attributed to miR-9^*^ are marked in red.

Abbreviations: ALL, acute lymphoblastic leukemia; AML, acute myeloid leukemia; BC, breast cancer; CA, cervical adenocarcinoma; CC, cervical cancer; CSCC, cervical squamous cell carcinoma; ΔEGFR, mutant epidermal growth factor receptor; ER, estrogen receptor; GBM, glioblastoma multiforme; HL, Hodgkin lymphoma; HM, hematological malignancies; MM, multiple myeloma; SCC, squamous cell carcinoma; TNBC, triple-negative breast cancer; WM, Waldenström macroglobulinemia.

Recently, several miRNA-based therapeutics have entered clinical trials in humans, e.g. miR-122 and miR-155. [96–100] As demonstrated in this review, miR-9/9^*^ may exert gross functional effects and change cellular phenotypes. The use of such miRNAs in human-cancer therapy might theoretically attenuate oncogenic effects and offer potential novel therapeutic avenues for treatment of human cancer. The precise functional role of miR-9/9^*^, however, depends on a specific cellular context and may consequently vary in different cell populations within one malignancy. Moreover, the capacity of miR-9/9^*^ to impact tumor formation does not necessarily predict their influence on the metastatic potential of tumor cells. These facts make future miR-9/9^*^-based anticancer therapies challenging. Furthermore, the potency of miR-9/9^*^ requires careful toxicity studies complemented with development of reliable and safe delivery methods to specifically target distinct cancer cell populations with miRNA mimics or antimiRs. Only when these technical issues are adequately addressed and we have a better understanding of miR-9/9^*^ biology both in health and disease, we can consider the full therapeutic potential of these miRNAs.
